# RETRACTED: Chalen-Moreano et al. Exploring the Antimicrobial Efficacy of Low-Cost Commercial Disinfectants Utilized in the Agro-Food Industry Wash Tanks: Towards Enhanced Hygiene Practices. *Foods* 2024, *13*, 1915

**DOI:** 10.3390/foods14142499

**Published:** 2025-07-17

**Authors:** Francisco Chalen-Moreano, Angélica Saeteros-Hernández, Paula Abdo-Peralta, Catherine Frey, Lilia Ofir Peralta-Saa, Andrea Damaris Hernández-Allauca, Carlos Rolando Rosero-Erazo, Theofilos Toulkeridis

**Affiliations:** 1 Faculty of Public Health, Escuela Superior Politécnica de Chimborazo, Km 1 ½ Panamericana Sur, Riobamba 060155, Ecuador; francisco.chalenm@espoch.edu.ec (F.C.-M.); asaeteros@espoch.edu.ec (A.S.-H.); l_peralta@espoch.edu.ec (L.O.P.-S.); 2 Independent Researcher, Riobamba 060155, Ecuador; paulitag_abdo@hotmal.com (P.A.-P.); carlos.rosero1733@gmail.com (C.R.R.-E.); 3 Faculty of Natural Resources, Escuela Superior Politécnica de Chimborazo, Km 1 ½ Panamericana Sur, Riobamba 060155, Ecuador; andrea.hernandez@espoch.edu.ec; 4 School of Geology, Aristotle University of Thessaloniki, 54124 Thessaloniki, Greece; ttoulkeridis@espe.edu.ec

The journal has retracted the article “Exploring the Antimicrobial Efficacy of Low-Cost Commercial Disinfectants Utilized in the Agro-Food Industry Wash Tanks: Towards Enhanced Hygiene Practices” [1].

Following publication, the Editorial Office was made aware of concerns regarding the use of a dataset included in the article [1].

In accordance with our complaints procedure, an investigation was conducted by the Editorial Office and the Editorial Board that, following input from the authors and representatives from the research institution CEBAS-CSIC, confirmed that the documented institutional authorization had not been obtained prior to publication. Despite the efforts of the authors, retroactive permission could not be obtained. As a result, the Editorial Board has decided to retract the article [1] in accordance with MDPI’s retraction policy (https://www.mdpi.com/ethics, accessed on 1 June 2025).

This retraction was approved by the Editor-in-Chief of *Foods*.

Francisco Chalen-Moreano agrees to this retraction; the remaining authors did not provide a comment on this decision.
